# Evaluating care pathways in Alzheimer’s disease: a qualitative interview study with GPs in England

**DOI:** 10.1136/bmjopen-2025-105978

**Published:** 2025-09-23

**Authors:** Mary Carter, Joanne E Butterworth, Chris Fox, Louise Allan

**Affiliations:** 1 Health and Community Sciences, University of Exeter Medical School, Exeter, UK; 2 Psychological Sciences, University of East Anglia Norwich Medical School, Norwich, UK

**Keywords:** Dementia, Primary Care, QUALITATIVE RESEARCH, Health Services

## Abstract

**Abstract:**

**Aim:**

To understand general practitioners’ (GPs’) experience of existing care pathways for people with moderate-severe Alzheimer’s Disease (AD) and explore their attitudes towards potential modifications to these pathways.

**Design:**

Secondary thematic analysis of qualitative interviews, originally conducted with GPs to explore prescribing of memantine in general practice. The theoretical domains framework was used to structure the data.

**Setting:**

The study participants were recruited via an online survey completed by GPs across England.

**Participants:**

Semi-structured, qualitative interviews were conducted with thirteen male and ten female GPs from a range of general practices in England.

**Primary outcome:**

Insights into GPs’ views and experiences regarding existing and possible care pathways for individuals with moderate to severe AD.

**Results:**

Gaps in GPs’ current levels of knowledge and skill in respect of caring for patients with moderate-to-severe AD affect their confidence and ability to identify opportunities for additional treatments. While GPs emphasise their role as providers of holistic care, features of the current healthcare context, including a lack of additional funding, inhibit their willingness to assume additional responsibilities as part of a revised pathway.

**Conclusion:**

A considerable knowledge, skills and confidence gap must be addressed to support the implementation of new care pathways that include revised responsibilities for GPs. GPs need appropriate support and resources to manage their patients’ changing needs and to provide the best possible pharmacological management as the disease develops.

**STRENGTHS AND LIMITATIONS OF THIS STUDY**

- Participants were recruited via an online survey, reaching general practitioners (GPs) in all English regions and with varied patient populations.
- Our analysis uniquely applies the theoretical domains framework to understand GP views on current care pathways for moderate to severe Alzheimer’s disease and how they may be enhanced.
- Clinicians working solely in secondary care/memory services were not included in our investigation.

## Background

 Dementia affects an estimated total of over 670 000 people in the UK, comprising 1 in 14 people over the age of 65 and 1 in 6 people over 80.^1^ The total number is projected to increase to 1.4 million by 2040. Dementia is the leading cause of death in the UK and resulted in over 75 000 deaths in 2023.^2^

Alzheimer’s Disease (AD) is the most common cause of dementia, accounting for up to 70% of diagnosed cases. It is a progressive disease, characterised by a range of cognitive and behavioural symptoms.^3^ The Clinical Dementia Rating Scale (CDR)^4^ is a global assessment tool used to gauge AD severity and guide treatment and medication choices. In trials, CDR scores of 1, 2 and 3 correspond to mild, moderate and severe AD, respectively. In 2020, the Alzheimer’s Society reported that 28% of cases in the UK were moderate and 14% were severe.^5^

Current treatments for AD recommended by the National Institute for Health and Care Excellence (NICE) include acetylcholinesterase inhibitors (AChEIs) prescribed individually to manage mild-to-moderate AD, and memantine, which is used in moderate-to-severe AD. AChEIs (eg, donepezil, galantamine and rivastigmine) inhibit the enzyme which normally breaks down acetylcholine, resulting in increased concentration in the brain, aiding communication between nerve cells and potentially providing temporary symptom relief or stabilisation. Memantine, an N-methyl-D-aspartate receptor antagonist, prevents excessive glutamate from damaging nerve cells in the brain^6^ and has demonstrated small improvements in cognitive and functional symptoms.^7 8^ When added to an AChEI, memantine may slow cognitive decline and improve behavioural and mood symptoms in moderate-to-severe AD.^9^ It is generally well-tolerated, though side effects can include drowsiness, dizziness, headaches, constipation and shortness of breath. Combined with donepezil, it may extend life expectancy by up to 5 years.^10^

New drugs, for example, lecanemab, have recently been approved in the USA, but draft guidelines from NICE^11^ recommend they are too expensive for use in the NHS. Even if approved in the UK in the future, only a minority of people with early AD and mild cognitive impairment would be eligible for treatment. With a lack of new disease-modifying drugs on the horizon, it is likely that AChEIs and memantine will continue to be used in the UK.

GPs are the first point of contact for people with suspected cognitive impairment or dementia in the UK. Current care pathways advise GPs to refer patients to memory clinics for initial assessment by specialists (psychiatrists, geriatricians, neurologists) early in the disease course. At these clinics, patients are commonly prescribed AChEIs in accordance with current (2018) NICE guidelines.^12^ Once discharged from secondary care, many patients continue to be monitored by primary care. According to the 2018 NICE guidelines, memantine may be initiated by a primary care doctor when a patient progresses to the moderate-to-severe stage of AD, if the patient is already taking an AChEI. In many local formularies in England, however, memantine is considered suitable for prescribing in primary care only after specialist initiation or recommendation, usually within a shared care protocol.

Our recent mixed methods research study^13^ adopted a pragmatic approach, employing both a survey and qualitative interviews^14^ to investigate GPs’ experience with the 2018 NICE recommendation and barriers to its implementation. Many GPs reported that they were unaware of the 2018 guidance. We found that local prescribing formularies were not supporting GPs in prescribing memantine, leading to low levels of confidence and expertise. GPs were uncertain about how to assess the severity of AD, which is crucial for deciding when to initiate memantine treatment. Owing to the absence of specific guidance or support from NICE, modifications to prescribing pathways for moderate-to-severe AD have not been widely implemented by local formularies or communicated to general practices.

While published literature lacks data on memantine prescribing trends following the 2018 NICE guideline, our analysis of the English Prescribing Dataset^15^ and prevalence data^16^ suggests that approximately 40% of individuals in England diagnosed with AD or mixed AD are receiving memantine, despite up to 70% being potentially eligible. This discrepancy implies that many people with moderate to severe AD may be missing out on optimal pharmacological treatment and face inconvenient and unnecessary referral to secondary care. Furthermore, potential cost savings associated with managing AD patients in one healthcare setting may be lost. We were therefore keen to further investigate this substantial treatment gap and assess GPs’ attitudes towards possible changes to existing care pathways which may address it.

Qualitative secondary analysis involves revisiting data from a previous study to explore important concepts that were not fully addressed in the original analysis, thereby enhancing the overall use and value of the data.^17 18^ In this secondary analysis, we re-examined interview transcripts and applied well-established constructs from the Theoretical Domains Framework (TDF)^19^ to explore GPs’ experiences and perceptions in respect of existing pathways for dementia care and identify ways in which these may be adapted to provide improved care for people with AD. The TDF is a validated framework which integrates constructs from several theories of behaviour.^20^ It is typically used to facilitate the systematic exploration of attitudes and experiences in respect of processes, pathways and guidelines.

For the secondary analysis, the research questions were:

From the perspective of GPs:

1. What are the positive and negative aspects of current care pathways for people with AD in England?
2. How may current care pathways in England be adjusted to provide improved care for people with AD?

## Method

We conducted a secondary qualitative analysis drawing on qualitative data from a mixed-methods study conducted from February to September 2024. The original study, comprising an online survey and semi-structured interviews, was approved by the University of Exeter Medical School and Health & Care Professions Ethics Committee (reference number 4302644).

### Study design and setting

Potential interviewees for the original study were identified through participation in an online survey of GPs in England. The survey gathered data on respondent demographics, their general practice and their experience of prescribing and care for patients with AD. Survey respondents who provided a contact email address at the end of the survey were sent further information, and arrangements for an online or telephone interview were made. A target sample matrix was developed to guide recruitment of a varied sample of GPs working in England.

### Data collection, management and analysis

MC, a non-clinical researcher, was responsible for obtaining informed consent from all participants and conducting all interviews. Interviews focused on GPs’ experiences of caring and prescribing for patients with AD, knowledge of memantine and current NICE guidance, confidence about assessing stage of disease in AD patients, support and training needs and working environment. The interview topic guide is included in online supplemental material (i).

MC transcribed all interviews verbatim and conducted a detailed review of each before creating an initial codebook of provisional codes based on the data.^21^ Transcripts were coded using QSR NVivo v14. JEB, an academic GP, independently reviewed and coded a subset of the transcripts. The two researchers discussed their coding and collaboratively developed a thematic framework. Themes were refined through ongoing discussion to ensure they accurately captured content and meaning within the data. Throughout the process, both researchers engaged in reflexive practice^22^ to minimise the influence of personal assumptions and biases.

For the secondary analysis of interview data, MC reassessed transcripts for relevance to the additional research questions, applied additional codes and mapped results to the TDF.

This report conforms to the Standards for Reporting Qualitative Research (SRQR).^23^ A completed SRQR checklist is included in the online supplemental material (ii).

### Patient and public involvement and engagement (PPIE)

Three carers and one person with mild dementia were recruited to a study PPIE group via study links with Innovations in Dementia,^24^ a not-for-profit, community interest company. PPIE members attended three online meetings, at which modifications to the interview topic guide and interpretation of the results were discussed.

## Results

Twenty-three interviews were conducted, 17 online and six by telephone. Interview length ranged from 29—54 min (mean 29 min). Interviewees’ details are presented in table 1:

**Table 1:** Details of interviewees

GP id	Gender	English region	Years as GP ≤10 >10	Practice list size <5000 5000–≤10 000 >10 000	IMD level of deprivation* ≤5 >5
GP_001	**M**	East	>10	*Locum GP*	
GP_002	**M**	London	>10	<5000	≤5
GP_003	**M**	West Midlands	≤10	>10 000	>5
GP_004	**M**	South West	>10	5000–≤10 000	>5
GP_005	**F**	South West	≤10	>10 000	>5
GP_006	**F**	London	≤10	*Local authority GP*	
GP_007	**M**	East	>10	>10 000	>5
GP_008	**M**	South West	>10	5000–≤10 000	>5
GP_009	**F**	North West	>10	>10 000	≤5
GP_010	**M**	West Midlands	>10	5000–≤10 000	>5
GP_011	**M**	East	>10	>10 000	≤5
GP_012	**M**	North East	≤10	5000–≤10 000	≤5
GP_013	**F**	North West	>10	>10 000	>5
GP_014	**F**	North West	>10	5000–≤10 000	≤5
GP_015	**M**	North West	>10	<5000	≤5
GP_016	**M**	North West	>10	5000–≤10 000	≤5
GP_017	**M**	Yorkshire & Humberside	≤10	>10 000	≤5
GP_018	**F**	North West	>10	*Not known*	≤5
GP_019	**M**	Yorkshire & Humberside	>10	5000–≤10 000	≤5
GP_020	**F**	North East	≤10	>10 000	≤5
GP_021	**F**	South East	>10	>10 000	≤5
GP_022	**F**	South East	≤10	>10 000	>5
GP_023	**F**	South East	>10	>10 000	≤5

* IMD deprivation decile (24) of general practice (lower number indicates greater deprivation).

Nine theoretical domains framework domains were considered relevant in our analysis and these are presented as themes below.

Online supplemental box 1 comprises theoretical domains, an explanatory description, example constructs and sub themes from our secondary analysis. Box 1 is included in online supplemental material (iii).

### Knowledge and skills applied to care of moderate-to-severe AD

Although separate domains in the TDF framework, knowledge and skills are closely related and presented together in our analysis.

The level of knowledge and skills concerning the care of patients with moderate-to-severe AD was variable among the interviewees. Many GPs had little experience of prescribing memantine and were not confident about assessing the stage of disease at which it may be helpful:

I think it is quite hard … you know, making those (dementia) stages so distinct, I think we’re very much usually dependent on our secondary care colleagues to tell us that that is the case. GP_023

Although GPs were confident that their interpersonal skills equipped them for understanding the impact of AD on patients and their families, many identified dementia care as a gap in their knowledge and skills, which should be addressed before introducing changes to existing care pathways and the division of responsibilities.

### Memory, attention and decision processes involved in care for moderate-to-severe AD

Evidence for this domain overlaps with skills and knowledge and focuses on retaining knowledge, filtering relevant information and selecting from several options.

In the context of caring for patients with moderate-to-severe AD, this domain relates to the mental capacity required by GPs for assessing patients with AD for their disease stage, and using this information to identify treatment options for patients with moderate-to-severe AD. In the absence of further training, these GPs were not ready for a change in the care pathway and would prefer for specialists to retain this responsibility:

I know that there’s been guidance about GPs being able to diagnose dementia without requiring a specialist assessment… we always refer, but that*’*s, I guess, because we haven’t been trained in doing it. GP_007

### Beliefs about capabilities in respect of care for moderate-to-severe AD

Beliefs about capabilities are closely linked with knowledge and skills applied to care of patients with moderate-to-severe AD, and this domain focuses on GPs’ confidence.

Several GPs believed that their role in current care pathways for dementia had diminished their expertise and undermined their confidence:

I think that because it’s something that has been done in secondary care … I suppose that GPs, they lack confidence… You become quite quickly deskilled… You know that it’s just been managed by secondary care for such a long time. GP_022

Many interview participants spoke of their reliance on specialist and community support in respect of caring for patients with AD:

In our practice, we’re really fortunate that we’ve got a really good system of social prescribing. We’ve got a well-being coordinator with an interest in elderly and mental health issues. So I would be keen to sort of get them (patients) hooked into one of those services. GP_019

Online and in-person sources of AD support for both GPs and patients were unevenly distributed, however, and some GPs reported feeling frustrated by their absence.

### Beliefs about consequences of modifying current GP responsibilities for moderate-to-severe AD

This domain encompasses worries expressed by some GPs with respect to initiating treatments for AD.

GPs expressed uncertainty about when to consider introducing memantine, how to evaluate its effectiveness and anticipate possible contraindications:

It’s not like starting a statin and looking at what reduction in cholesterol there is. It’s much more nuanced than that, isn’t it? GP_004

Some GPs believed that, with additional training and support, they could participate in a revised pathway to provide improved care for patients:

(Patients) will be… quite quickly discharged and there are lots of those patients and it’s very hard sometimes to re-refer them back… to get them back in that clinic and be seen by an old-age psychiatrist or a neurologist… So I think for lots of reasons… it probably would be very good for us to be more upskilled. GP_011

### Alignment of social professional role and identity with care pathways

This domain focuses on how an individual clinician’s sense of professional or social identity guides their behaviour.

GPs invariably viewed holistic care as an essential part of their role in caring for individuals with AD and their families. They emphasised the importance of non-pharmacological approaches to care and suggested that this essential aspect of care was provided only in general practice:

I think the main (thing) is the centrality of holism, generally and sometimes the difficulties I have with specialists are that people are in their silos, so the psychiatrist is focused on just psychiatry and the geriatricians… it sometimes feels like me and my nurse colleague are the only ones that are taking the whole patient into account, and I think that’s a really valuable perspective. GP_012

Some accepted that responsibility for diagnosis and initiation of treatment for AD would become part of their professional role:

I’d see us being able to start treatment earlier. Possibly make a better benefit for the patient and give them a better quality of life for longer, which I think is the only thing we can actually ask for. And yeah, the ongoing relationship is there… I think it’s a natural progression for GPs to be able to take that on. GP_009

GPs at times voiced concern about colleagues who believed that diagnosing dementia should be left to specialists, pointing out that attendance at a memory clinic was not appropriate for all patients:

I even when they see GPS not diagnosing dementia, when it’s clear that that’s the diagnosis, they feel that that’s the memory service’s job. But you get people who aren’t really fit or able…, who clearly have dementia but aren’t being diagnosed. GP_003

### Impact of environmental context and resources on care pathways

This domain focuses on the external and resource-based factors that influence behaviour.

Current guidance from local formularies usually supports prescribing memantine in primary care only after it has been initiated by a specialist. GPs invariably spoke of long waits for patients between referral from general practice and an appointment with memory services and found this situation unsatisfactory for a vulnerable patient group. Some believed that a change to the existing care pathway would be helpful:

Nightmare situation that we have… a service that takes months to get an appointment… then eventually, after about 6 months after the referral, we get a diagnosis. I don’t see why GPs can’t do it. GP_009

Some GPs believed that existing care pathways took insufficient account of patients’ home circumstances and the importance of involving familiar healthcare staff throughout the patient’s journey:

If you send someone to a memory clinic, in my experience… there was quite a narrow look at what the issues were. And often I saw my role as to work with family and carers and the patients, think about ‘what are the range of issues going on here?*’* GP_018

Although GPs recognised the importance of continuity of care for this patient group, they were not always able to provide it:

But sadly, the situation in general practice is not that straightforward. There might not be an appointment for maybe a month, things might have got worse before then… So yes, we want continuity… and sometimes it does happen, but sometimes… the patients then go to other people. GP_020

Many GPs reported that there was insufficient time in general practice for thorough Assessments of patients with AD, and opportunities for patients to book double or extended appointments were sometimes limited:

I think that’s the challenge of general practice. We just haven’t got the time to be doing bigger memory things in order to try and make that diagnosis. GP_008

Some GPs were satisfied with the existing care pathway and division of responsibilities between specialist and primary care, believing it enabled each to work to their respective professional strengths:

I think they come back from memory clinic well-informed, and you know they’ve got a diagnosis, and they’ve got a plan, and they’ve spoken to somebody, and… a lot of people feel quite relieved. And certainly the families do. I think the experience of coming back into primary care has been a good one. GP_013

In contrast, several GPs identified potential benefits in adjusting responsibilities for this group of patients:

I’m starting to think, if the guidance is… more accepting of prescribing and diagnosing in the community, except in those special cases where you’re looking at the other dementias, or where people might need imaging of some sort or a further test. Then I’m not sure there’s a huge scope for those (memory clinics) in the future, given the safety and the cost of the drugs. GP_016

In some areas, care pathways varied from the standard model. One service was designed to support dementia patients at home and reduce hospital admissions, while elsewhere GPs with extended roles in dementia (GPwERs) worked across memory services and general practice, boosting system capacity and their own confidence in managing dementia:

So there is probably a shorter wait for me and the waiting list has dropped since the GPs have been working in the memory service… I also work in the frailty hub, which is like an MDT and I lead that for the practice… I am quite happy with signposting (eg, to Admiral nurses, AgeUK dementia coordinators) because I work with them so closely and I probably am the link person in the surgery for other GPs… I’m much more confident now than I was before I did this role. GP_022

### Social influences impacting attitudes towards AD care pathways

This domain overlaps with ‘Environmental context’ described above and relates to the influence on GPs of their general practice culture and the wider healthcare environment.

Many GPs worked collaboratively with their general practice colleagues to optimise care for patients with AD:

We have a weekly MDT where we discuss cases that are challenging or we’ve got concerns over… We have set time where we talk about patients who we feel have either safeguarding risks or are starting to come into our sort of sphere of awareness, so that we’re all aware of it, because none of us are 5 days a week. GP_016

Although some GPs reported working well with specialist care teams in current care pathways, many were critical of their relationship with memory services:

I think the thing that would help most would be having more integration and support from the dementia support team at the Older Persons Mental Health Service, which is very… feels quite distant. I think that’s probably the thing that would help most. GP_007

### Reinforcement of beliefs about existing and future care pathways

This domain encompasses incentives and rewards and how they impact attitudes towards existing or future care pathways.

The absence of additional funding and resources influenced GPs’ attitudes towards reconfiguration of care pathways for patients with dementia. In particular, collaboration with colleagues in primary care networks to provide care for patients with AD was not perceived as beneficial unless backed by increased funding and staffing:

I think the idea that joining everyone together without extra resources is going to release all these efficiencies… I'm not convinced by that. GP_012

Many GPs believed that modifications to existing pathways, which included additional responsibilities for GPs were contingent on extra training as well as increased funding:

I think what we need is almost like GPs with extended specialist roles that can sort of pull some of that work out of secondary care and be that buffer of support… but again, that’s all about funding and it’s all about upskilling and frameworks. GP_023:

The remaining five TDF domains (Behavioural regulation, Intentions, Goals, Emotion, Optimism) are not discussed here, as references to relevant constructs were not identified in our analysis. These remaining domains may be included in future work.

## Discussion

### Principal findings

This secondary analysis used the lens of the TDF to investigate GPs’ perceptions of current care pathways and responsibilities in respect of patients with AD and the potential for modification.

We have identified nine TDF domains which are relevant to this issue. When considering potential modifications to pathways, many GPs have concerns about their current levels of **Knowledge** and **Skills** in respect of AD, and consequently their ability to map AD stages to opportunities for further prescribing (**Decision processes**). These skills and knowledge gaps are linked with GPs’ confidence in caring for moderate-to-severe AD (**Beliefs about capabilities**) and trust in memantine’s effectiveness and possible contraindications (**Beliefs about consequences**). Although a few GPs believe that patients with moderate to severe AD derive benefit from current care pathways, and from the existing balance of responsibilities, skills and knowledge between general practice, and specialist teams, this is not a typical view and many believe that the current situation results in delays for patients. GPs identify features of the current healthcare context, such as relationships within general practices and primary care networks, and with specialist care teams, which variously impact their capacity to provide optimal care for patients with AD (**Environment, Social influences**). GPs emphasise their professional ability to provide holistic care to AD patients and their families, with whom they have fostered ongoing relationships (**Social/Professional role**), but many believe that a lack of additional funding is a barrier to modifying their responsibilities for AD (**Reinforcement**).

### Strengths and limitations

This study reports one of the few investigations into GPs’ perspectives on care pathways for AD, and was undertaken in response to an apparent lack of engagement with recent revisions to UK national guidance regarding the management of patients with moderate-to-severe AD. The data are based on interviews with GPs working in locations throughout England, in a range of socio-economic settings and in practices with varied characteristics. This secondary analysis builds on our earlier work to provide a comprehensive understanding of the characteristics of current care pathways for AD and the potential for modification.

The findings presented in this study reflect the views and experiences of participants who chose to take part and may have done so due to a specific interest in dementia. While our findings include insights from two GPs with a dual role in both primary and secondary care, obtaining the perspectives of clinicians working solely in memory services was beyond the scope of this investigation.

### Comparison with existing literature

This is a unique study of GPs’ perceptions about existing and potential care pathways in England for patients with moderate-to-severe AD. Mapping to the theoretical constructs of the TDF has been used previously to explore behavioural determinants associated with care for the elderly,^25 26^ diagnosis and management of dementia^27^ and preparation of handover information for hospital discharge.^28^ In our analysis of GPs’ views about patient pathways and the division of responsibilities between general practice and specialist services, we have found that some of the same TDF domains identified in our analysis were relevant to these previous studies. Cadogan *et al*^25^ and Murphy *et al*^27^ identified a **Knowledge/Skills** gap in respect of guidelines for prescribing for patients with dementia, and we found similar concerns about prescribing memantine for patients with moderate-to-severe AD. Participants in Markiewicz *et al*’s study^28^ were self-assured about specific professional **skills**, and this confidence (**Belief in capability**) was echoed by the GPs in our study, who linked their interpersonal skills with their particular contribution to the care pathway (**Professional role**). Many GPs in our study were reluctant to assume an augmented role in the patient pathway in the absence of additional funding (**Reinforcement**), but this domain was not predominant in previous literature.

Our findings are consistent with a 2017 survey, conducted in 25 countries, which examined primary care physicians’ (PCPs) attitudes towards dementia management.^29^ The survey found that PCPs who are allowed to prescribe dementia-related treatments (**Skills, Professional role**) were more inclined to assume additional responsibilities in managing their patients’ care. As with the 2017 survey, GPs in our study reported a lack of time for thorough assessment of patients with AD (**Environment**), leading to a continued reliance on memory services.

As with our analysis, data in previous studies have been assigned to more than one TDF domain,^30 31^ reflecting the interconnectedness of the theoretical constructs. A recent investigation of decarbonisation in general practice,^32^ which assigned data to constructs from both the TDF and normalisation process theory,^33^ suggests that mapping to multiple domains (within each framework) may allow for a deeper and more reliable interpretation of the data, while also mentioning the potential for a reduction in precision.

### Implications for practice and research

Exploration of the key drivers and challenges associated with modifying current pathways for dementia has identified several messages for local healthcare decision-makers, including those responsible for devising formularies and general practices. A considerable knowledge, skills and confidence gap is identified by GPs, and many believe that this must be addressed if additional responsibilities for AD are to be assumed in general practice. While there is interest in modifying the care pathway for patients with moderate-to-severe AD, GPs require appropriate and timely support to optimise their ongoing relationships with patients so that they can recognise opportunities for introducing additional treatment options. GPs value the ongoing support they receive from community teams, multi-disciplinary groups and specialists, including online ‘advice and guidance’;^34^ new care pathways should actively incorporate and promote these beneficial features, particularly in respect of the management of less common dementias. The deployment of GPwERs, shared between general practice and memory services, may be an important component of an improved model of care, which enhances capacity in the system and confidence in respect of diagnosis and treatment of AD in general practice settings.

## Conclusion

The misalignment between current NICE guidelines and local prescribing advice needs to be resolved, as it contributes to uncertainty and delay within the system. There is a considerable gap in knowledge, skills and confidence which must be bridged to support the effective implementation of new care pathways. GPs need appropriate support and resources to respond to their patients’ evolving needs and to deliver the best possible pharmacological care as conditions progress.

## Supplementary material

10.1136/bmjopen-2025-105978 online supplemental file 1

10.1136/bmjopen-2025-105978 online supplemental file 2

10.1136/bmjopen-2025-105978 online supplemental file 3

## Data Availability

Data are available upon reasonable request.
