# Supplementary material for: Evaluating care pathways in Alzheimer’s disease: a qualitative interview study with GPs in England
Source: BMJ Open. 2025 Sep 23;15(9):e105978. doi: 10.1136/bmjopen-2025-105978 (PMC12458784; doi:10.1136/bmjopen-2025-105978)
Supplement: online supplemental file 1 [file bmjopen-15-9-s001.docx]

**Box 1: Theoretical (TDF) domains, descriptions & sub themes**

| **TDF DOMAIN** | **KNOWLEDGE & SKILLS applied to care of moderate-to-severe AD** | | **MEMORY, ATTENTION & DECISION PROCESSES involved in care for moderate-to-severe AD** | **BELIEFS ABOUT CAPABILITIES in respect of care for moderate-to-severe AD** | **BELIEFS ABOUT CONSEQUENCES of modifying current GP responsibilities for moderate-to-severe AD** | **Alignment of SOCIAL PROFESSIONAL ROLE & IDENTITY with care pathways** | **Impact of ENVIRONMENTAL CONTEXT & RESOURCES on care pathways** | **SOCIAL INFLUENCES impacting attitudes towards AD care pathways** | **REINFORCEMENT of beliefs about existing and future care pathways** |
| --- | --- | --- | --- | --- | --- | --- | --- | --- | --- |
| **Description *& example construct*** | An awareness of the existence of something:  *Knowledge about a condition Procedural knowledge* | An ability or proficiency acquired through practice:  *Competence Interpersonal skills* | The ability to retain information, focus selectively on aspects of the environment & choose between two or more alternatives:  *Decision-making Cognitive overload* | Acceptance of the truth, reality or validity about an ability, talent or facility that a person can put to constructive use:  *Self-confidence Perceived competence* | Acceptance of the truth, reality or validity about outcomes of a behaviour in a given situation:  *Beliefs Outcome expectancies* | A coherent set of behaviours & displayed personal qualities of an individual in a social or work setting:  *Professional role Professional boundaries* | Circumstances of a situation or environment that discourages or encourages the development of skills & abilities, independence, social competence & adaptive behaviour:  *Resources Organisational culture* | Those interpersonal processes that can cause individuals to change their thoughts, feelings or behaviours:  *Group norms Team working* | Increasing the probability of a response by arranging a dependent relationship, or contingency, between the response & a given stimulus:  *Rewards Incentives* |
| **SUB THEMES FROM ANALYSIS** | | | | | | | | | |
| Prescribing memantine |  |  |  |  |  |  |  |  |  |
| Evaluating disease impact |  |  |  |  |  |  |  |  |  |
| Interpersonal skills |  |  |  |  |  |  |  |  |  |
| Evaluating treatment options |  |  |  |  |  |  |  |  |  |
| Identifying disease stage |  |  |  |  |  |  |  |  |  |
| Availability of support |  |  |  |  |  |  |  |  |  |
| Confidence in self |  |  |  |  |  |  |  |  |  |
| Confidence in memantine |  |  |  |  |  |  |  |  |  |
| Perception of GP role |  |  |  |  |  |  |  |  |  |
| Local guidance for memantine initiation |  |  |  |  |  |  |  |  |  |
| Delays in current pathway |  |  |  |  |  |  |  |  |  |
| Lack of time in consultation |  |  |  |  |  |  |  |  |  |
| Pressure on appointments |  |  |  |  |  |  |  |  |  |
| Emphasis on pharmacological solutions |  |  |  |  |  |  |  |  |  |
| Relationships within practices & PCNs |  |  |  |  |  |  |  |  |  |
| Relationships with specialists |  |  |  |  |  |  |  |  |  |
| Funding to support change |  |  |  |  |  |  |  |  |  |
| Practice priorities |  |  |  |  |  |  |  |  |  |
