# Supplementary material for: Evaluating care pathways in Alzheimer’s disease: a qualitative interview study with GPs in England
Source: BMJ Open. 2025 Sep 23;15(9):e105978. doi: 10.1136/bmjopen-2025-105978 (PMC12458784; doi:10.1136/bmjopen-2025-105978)
Supplement: online supplemental file 2 [file bmjopen-15-9-s002.docx]

**TOPIC GUIDE FOR MEMANTINE INTERVIEWS**

**PRESCRIBING MEMANTINE IN GENERAL PRACTICE IN ENGLAND**

**Researcher:  Dr Mary Carter**

| **Question & prompt** |
| --- |
| 1. What is your experience of caring and prescribing for patients with AD?   *PROMPT*  *Explore challenges, differences between mild/moderate/severe AD, any special interest in dementia* |
| 1. Are you confident about deciding whether a patient has mild, moderate or severe AD?   *PROMPT*  *Do GPs and carers/patients have a shared definition of mild/moderate/severe? What might help you with identifying the severity of AD? Are the existing tools (MMSE, GPCOG etc.) helpful?* |
| 1. How difficult/easy is giving news of a possible AD diagnosis to patients and families?   *PROMPT Are you able to signpost patient/family to other sources of help so that they can make informed decisions? Are carers registered?* |
| 1. How often do you review patients with AD?   *PROMPT*  *Do you think this is enough? Do you review carers?* |
| 1. What is your experience of prescribing memantine for moderate to severe AD?   *PROMPT*  *Explore reasons for prescribing/not prescribing, eg time, cost, role of GP and specialist services, efficacy/safety/tolerability of memantine, prescribing memantine alone/in combination with cholinesterase inhibitors, other medications, do you discuss medications routinely with patients/carers?* |
| 1. How has your prescribing practice been impacted by NICE guidance NG97 about prescribing memantine in general practice for moderate to severe AD?   *PROMPT*  *Are you restricted by guidance from the local formulary? Explore awareness of current NICE guidance* |
| 1. What types of support or training could assist your care/prescribing for patients with moderate to severe AD?   *PROMPT*  *Access to advice; sources of information; guidelines, training topics and formats* |
| 1. How does your existing relationship with a patient affect your decision-making in respect of AD?   *PROMPT*  *Continuity of care with patient/family; no family support, mild/moderate/severe AD* |
| 1. In your experience, how is shared decision making impacted by AD?   *PROMPT*  *Differences according to severity (mild, moderate, severe); involving the patient’s carer, capacity issues* |
| 1. How does your working environment affect your care/prescribing for patients with moderate to severe AD?   *PROMPT*  *Time, availability of (double) appointments, practice culture, support from specialists within and outside practice (nb PCN), access to training, possibility of dedicated dementia clinics* |
| 1. Do you have any other comments about caring/prescribing for patients with moderate to severe AD? |
