# Supplementary material for: Evaluating care pathways in Alzheimer’s disease: a qualitative interview study with GPs in England
Source: BMJ Open. 2025 Sep 23;15(9):e105978. doi: 10.1136/bmjopen-2025-105978 (PMC12458784; doi:10.1136/bmjopen-2025-105978)
Supplement: Supplementary file 4 [file bmjopen-2025-105978.reviewer_comments.pdf]

PEER REVIEW HISTORY

BMJ Open publishes all reviews undertaken for accepted manuscripts. Reviewers are asked to complete a checklist review form and are provided with free text boxes to elaborate on their assessment. These free text comments are reproduced below.

ARTICLE DETAILS

Title (Provisional)

Evaluating care pathways in Alzheimer’s Disease: A qualitative interview study with GPs in England

Authors

Carter, Mary; Butterworth, Joanne E.; Fox, Chris; Allan, Louise

---

VERSION 1 - REVIEW

**Reviewer**	**1**
**Name**	**Lech, Sonia**
**Affiliation**	**Berlin Institute of Health Institute for Medical Sociology and Rehabilitation Science**
**Date**	**24-Jun-2025**
**COI**	**None**

Introduction:

The study rational in clear, and the literature well described. However, I am lacking the „new“ aspect of the study (and its results). In the last years lots of work looked on primary care and dementia and there is qualitative and quantitative data on the problems in the care. Why did you decide to study this topic further? What is the new aspect/theory/approach you were examining. Please, make sure you explain better the novelty of your study.

Methods:

Was ethical votum obtained for this study. I understand that for the main study yes. But did that also include the interviews? Please, clarify.

Discussion:

This section seems to be not in line with the standard reporting style of BMJ Open. Please, structure and re write this section accordingly. Further, the comparison of present results and past literature is rather short. I would like to read a more in depth discussion of present results, in particular because the novelty of this study remains unclear to me. Further, I miss some up to date references/Studies.

**Reviewer**	**2**
**Name**	**Ma, Richard**
**Affiliation**	**University of Bristol Faculty of Health Sciences, Centre for Academic Primary Care**
**Date**	**17-Jul-2025**
**COI**	**None**

Well written qualitative study into an important topic in primary care. I was not aware GPs could prescribe memantine in some circumstances, so I have learnt something myself, and aligns with the message you are highlighting.

There are a few points I wish to make which would help clarify the methods, findings and discussions.

The background was clear and seemed to lead to the aims and objectives. I think you were trying to use a framework to explain implementation of guidelines into clinical practice. I note the use of Theoretical Domains Framework - I read it as a framework rather than a theory in itself. The text implied it is a theory though. I think it needs a couple of sentences to explain why that was chosen over others such as Normalisation Process Theory or Theory of Planned Behaviour.

Method was well described and thematic analysis was conducted with reflexive practice to minimise influence of personal assumptions and bias. Reporting checklist included -SRQR.

Expand "TDF" in the Box 1 caption.

I found a few instances where data overlapped more than one domain. I suggest offer an explanation as to why that might be problematic; eg does it reflect insufficient data to put into discrete domains/themes? Where the themes/domains poorly defined or was the framework in appropriate for your data? Some of your interviews were 30mins - were they too short to get the data you needed?

Lastly, you had two questions in the beginning: 1. What are the positive and negative aspects of current care pathways for people with AD in England?

2. How may current care pathways in England be adjusted to provide improved care for people with AD?

I think you answered (2) adequately but I am not sure you explicitly answered (1).

VERSION 1 - AUTHOR RESPONSE

REVIEWER 1

6	Introduction:<br>The study rational in clear, and the literature well described. However, I am lacking the „new“ aspect of the study (and its results). In the last years lots of work looked on primary care and dementia and there is qualitative and quantitative data on the problems in the care.<br><br>Why did you decide to study this topic further? What is the new aspect/theory/approach you were examining. Please, make sure you explain better the novelty of your study.	We have edited the Background section to clarify the motivation for our research. It now highlights the disparity between national (NICE) guidance and the local advice followed by general practices in the UK and what this means for people with AD.<br><br>We have edited the Discussion section to more clearly articulate the appropriateness of the TDF and the underlying rationale for our study.	Background, paras 5 & 7, p4<br><br>*Strengths & limitations* paras 1 & 2, p10
7	Methods:<br>Was ethical votum obtained for this study. I understand that for the main study yes. But did that also include the interviews? Please, clarify.	We have amended and clarified the Methods paragraph about ethical review.	Methods para 1, p4
8	Discussion:<br>This section seems to be not in line with the standard reporting style of BMJ Open. Please, structure and re write this section accordingly.<br><br>Further, the comparison of present results and past literature is rather short. I would like to read a more in-depth discussion of present results, in particular because the novelty of this study remains unclear to me. Further, I miss some up to date references/Studies.	We have made changes to the Discussion to better reflect BMJ Open’s suggested format.<br><br>We have strengthened the first paragraph of the *Comparison with existing literature* section; it now includes references to more recent research. We have added a paragraph to this section, which considers the use of the TDF in our study within the context of past literature.	Discussion, pp 9-11<br><br>*Comparison with existing literature,* paras 1 & 3, p10
Reviewer 2			
9	Well written qualitative study into an important topic in primary care. I was not aware GPs could prescribe memantine in some circumstances, so I have learnt something myself, and	Thank you for the positive feedback.	

	aligns with the message you are highlighting.		
10	The background was clear and seemed to lead to the aims and objectives. I think you were trying to use a framework to explain implementation of guidelines into clinical practice. I note the use of Theoretical Domains Framework - I read it as a framework rather than a theory in itself. The text implied it is a theory though. I think it needs a couple of sentences to explain why that was chosen over others such as Normalisation Process Theory or Theory of Planned Behaviour.	We agree that TDF is a framework of constructs drawn from a range of theories and have edited the Discussion accordingly.	*Strengths & limitations* paras 1 & 2, p10
11	Method was well described and thematic analysis was conducted with reflexive practice to minimise influence of personal assumptions and bias. Reporting checklist included -SRQR.	Thank you for the positive feedback.	

12	Expand "TDF" in the Box 1 caption.	We have expanded 'TDF domains' to *'Theoretical (TDF) domains, descriptions and sub-themes'* in the Box 1 caption.	Results, (signpost to separate document) p5
13	I found a few instances where data overlapped more than one domain. I suggest offer an explanation as to why that might be problematic; eg does it reflect insufficient data to put into discrete domains/themes? Where the themes/domains poorly defined or was the framework in appropriate for your data?<br><br>Some of your interviews were 30mins - were they too short to get the data you needed?	We have added a paragraph to the Discussion, which contains references other studies in which data has been assigned to multiple TDF domains. We offer a justification for this, while also suggesting that researchers may need to be aware that this practice may result in less precise conclusions.<br><br>We were restricted by individual GPs' availability, which was often limited. The interview topic guide was sufficiently flexible to enable us to focus on our key research interests within the time available.	*Comparison with existing literature*, para 3, p10
14	Lastly, you had two questions in the beginning: 1. What are the positive and negative aspects of current care pathways for people with AD in England?<br>2. How may current care pathways in England be adjusted to provide improved care for people with AD?<br>I think you answered (2) adequately but I am not sure you explicitly answered (1).	We have edited the Discussion to include explicit mention of the positive and negative aspects of current pathways as perceived by GPs.	*Principal findings*, paras 1 & 2, p9

VERSION 2 - REVIEW

Reviewer 2

Name Ma, Richard

Affiliation University of Bristol Faculty of Health Sciences, Centre for Academic Primary Care

Date **03-Sep-2025**

COI

I am satisfied that the authors have addressed the reviewers' comments.

I wonder if the rationale for the use of TDF would sit better at the end of the Background section rather than in the Discussion.

Page 11 line 35 "Fleming et al (27)" is struck through but should be deleted.

VERSION 2 - AUTHOR RESPONSE

1. I have moved the rationale for using the Theoretical Domains Framework (TDF) to the end of the Background section on page 4. This has entailed some adjustments to citation numbers and references in the manuscript.
2. I have deleted the strike-through text ('Fleming et al [27]') from page 11.
